# Supplementary material for: Peritoneal tissue-resident macrophages are metabolically poised to engage microbes using tissue-niche fuels
Source: Nat Commun. 2017 Dec 12;8:2074. doi: 10.1038/s41467-017-02092-0 (PMC5727035; doi:10.1038/s41467-017-02092-0)

Supplementary Figure 1

Proportional abundances of macrophage metabolites are distinct and reveal significant enrichment of peritoneal tissue-resident macrophage TCA cycle metabolites

a) Heat-map showing the log10 difference from the average metabolite peak areas from Gas Chromatography-Mass Spectrometry of 363 metabolites between 3 independent peritoneal tissue-resident macrophage (Resident) and 6 independent bone marrow-derived macrophages (BMDM) extracts. **b)** these data were analysed with Ingenuity pathway analysis (IPA) for a non-biased view of enriched metabolic pathways, data shown is the minus log2 *p*-value. Tissue-resident macrophages were enriched for the putrescine biosynthesis pathway and TCA metabolites, while BMDM contained relatively higher levels of amino acids, characterized by enrichments of numerous pathways, but mainly transfer-ribonucleic acid (tRNA) charging (which occurs using amino-acids during protein synthesis). **c)** Heat-map showing the log10 difference from the average metabolite peak areas of amino acids from (a).

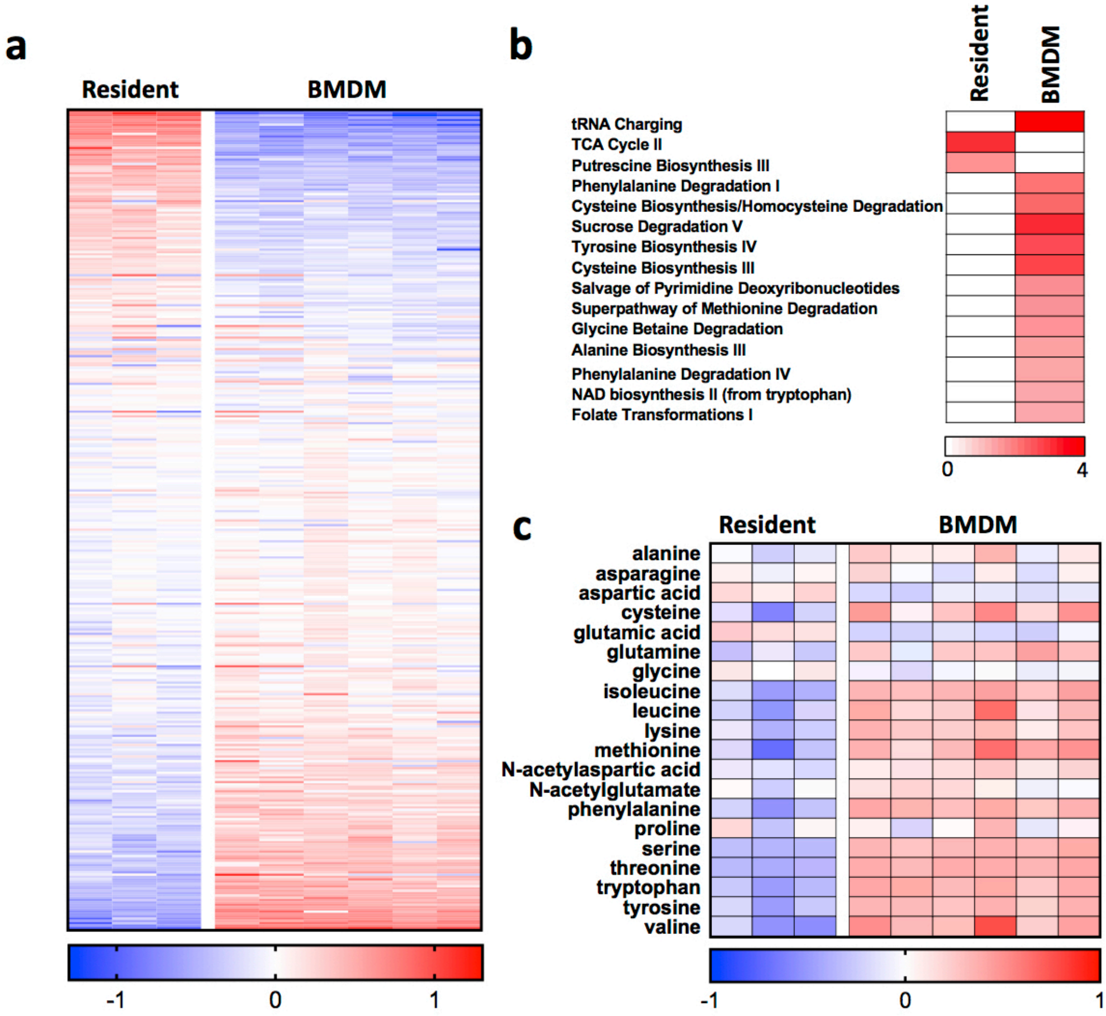
a
Resident
BMDM
-1
0
1
b
Resident
BMDM
tRNA Charging
TCA Cycle II
Putrescine Biosynthesis III
Phenylalanine Degradation I
Cysteine Biosynthesis/Homocysteine Degradation
Sucrose Degradation V
Tyrosine Biosynthesis IV
Cysteine Biosynthesis III
Salvage of Pyrimidine Deoxyribonucleotides
Superpathway of Methionine Degradation
Glycine Betaine Degradation
Alanine Biosynthesis III
Phenylalanine Degradation IV
NAD biosynthesis II (from tryptophan)
Folate Transformations I
0
4
c
Resident
BMDM
alanine
asparagine
aspartic acid
cysteine
glutamic acid
glutamine
glycine
isoleucine
leucine
lysine
methionine
N-acetylaspartic acid
N-acetylglutamate
phenylalanine
proline
serine
threonine
tryptophan
tyrosine
valine
-1
0
1

Supplementary Figure 2

Peritoneal tissue-resident and bone marrow-derived macrophage mRNA expression patterns show differential pathway regulation

Heat-map showing the differentially enriched pathways and Z-activation scores of two bone marrow-derived (BMDM) and two peritoneal tissue-resident macrophages (Res) RNA samples. Genes that were significantly >2-fold regulated in RNA-sequencing data from Res and BMDM were analysed with Ingenuity pathway analysis (IPA) to generate the dataset. A positive Z-activation score shows positive pathway regulation, while a negative score shows the opposite.

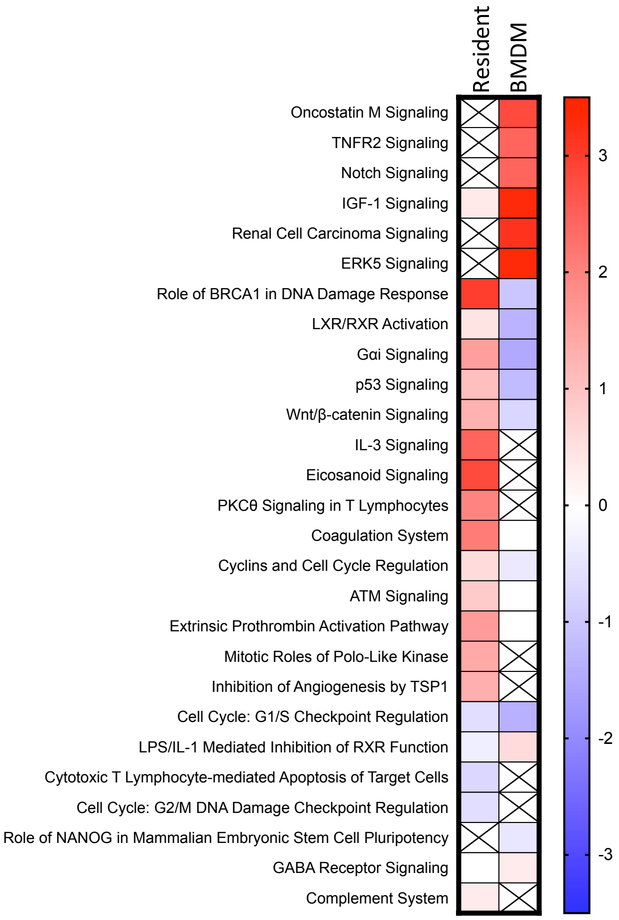

Supplementary Figure 3

Peritoneal tissue-resident and bone marrow-derived macrophages have differential expression of metabolic gene isoforms, but show no gross pathway differences

Heat-map showing the log10 difference from the average metabolic gene expression of two bone marrow-derived (BMDM) and two peritoneal tissue-resident macrophages (Res) RNA samples. The data reveal differential expression of different metabolic isoforms between Res and BMDM, although only minor differences in total mitochondrial complex and citric acid cycle gene expression.

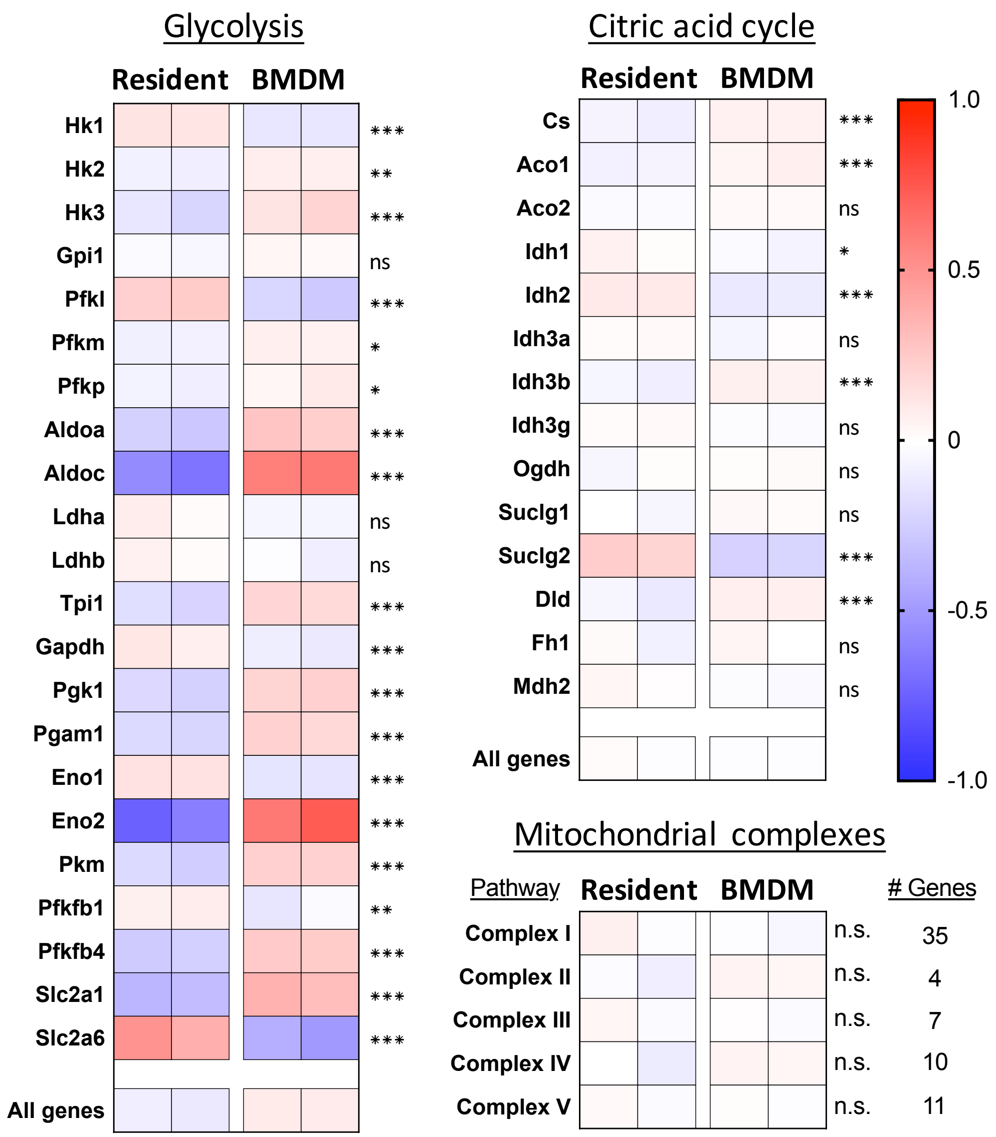

Supplementary Figure 4

Peritoneal tissue-resident macrophages have larger mitochondrial networks than bone marrow-derived macrophages

a) Scatter plot showing the mean mitochondrial volume of peritoneal tissue-resident (Res) and bone marrow-derived macrophages (BMDM) from at least 9 images, pooled from two independent observations (Res n=34, BMDM n=30). **b)** Bar graph depictions of the number of mitochondrial networks and mean volumes from a. **c)** Bar graph showing the total mitochondrial volume in individual cells combined from data in a. Data (b,c) were analysed by Student's t-test. Error bars=mean±SEM.

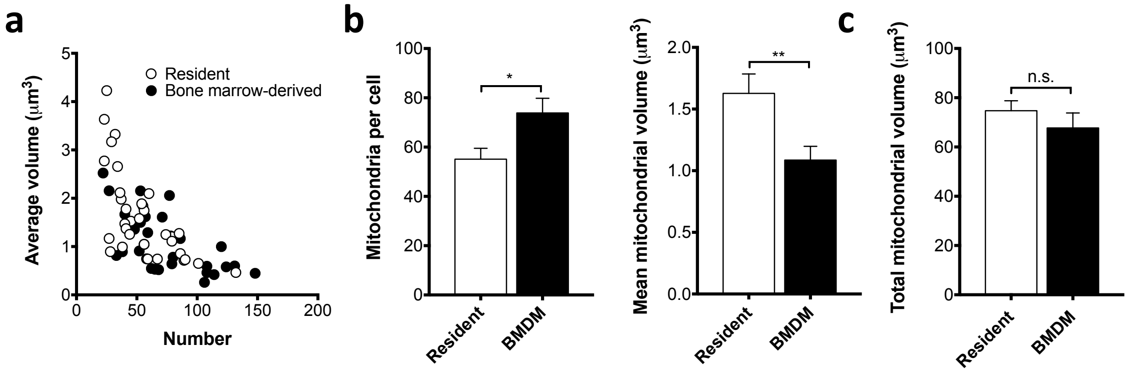

Supplementary Figure 5

Proportional abundances of serum and lavage metabolites are distinct and reveal significant differences between fatty acid and amino acid pathways

Heat-map showing the log10 difference from the average metabolite peak areas from Gas Chromatography-Mass Spectrometry of 189 named metabolites between 5 independent peritoneal fluid lavages and 5 independent serum extracts. These data were analysed with Ingenuity pathway analysis (IPA) for a non-biased view of enriched metabolic pathways (right), IPA data is shown as minus log2 *p*-value.

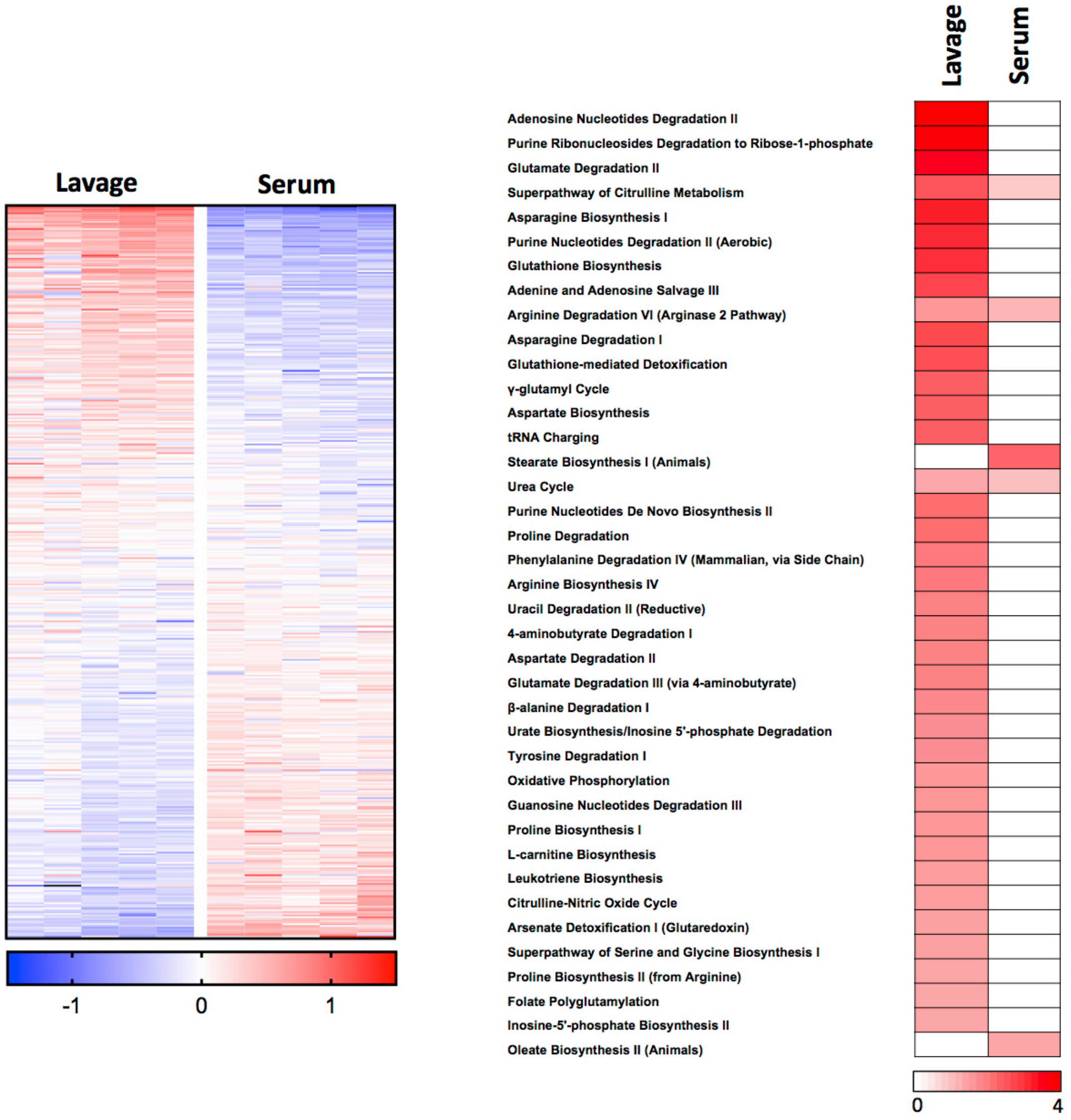

Supplementary Figure 6

Additional biological ligands can induce PKC-dependent, glutamine-dependent oxygen consumption in peritoneal tissue-resident macrophages

a) Plot of oxygen consumption rate (OCR) vs time in peritoneal tissue-resident macrophages (Res). Ligands were added after the first reading: *Saccharomyces cerevisiae* (*S.cer*, 50µg/ml, heat-killed + opsonized); *N*-Formylmethionyl-leucyl-phenylalanine (fMLF, 1µM); TLR1/2 ligand Pam3CSK4 (1µg/ml); Inflammasome-inducing uric acid crystals (50µg/ml); apoptotic thymocytes (500,000 cells); and the TLR7/8 ligand R848 (1µg/ml). **b)** Bar chart showing the percentage of total oxygen consumption induced by ligands. The values show the relative maintenance of induced oxygen consumption in the absence of glutamine (the values were normalized to 100% in the presence of glutamine). **c)** Bar chart showing the percentage of total oxygen consumption induced by ligands. The values show the maintenance of ligand-induced oxygen consumption after a 1h pre-treatment with the protein kinase C (PKC) inhibitor sotrastaurin (5µM) (the values were normalized to 100% with vehicle control). Data are pooled from 2-4 independent experiments (b: n=7-14 c: n=13-20) and were analysed by two-way ANOVA with depicted Sidak's post-tests; (b): interaction p=0.2883, different stimuli p=0.2527, effect of glutamine p<0.0001; (c): interaction p=0.0781, different stimuli p=0.0316, effect of PKC inhibition p<0.0001. All error bars denote mean±SEM.

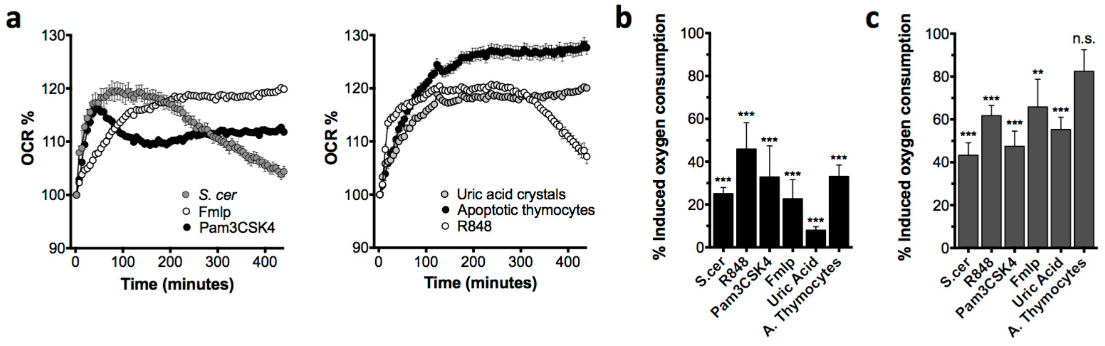

Supplementary Figure 7

Bone marrow-derived macrophages do not burst in response to zymosan

a) Representative density plot showing bone marrow-derived macrophage (BMDM) uptake of pHrodo zymosan particles (50µg/ml) after 1h. Data are from one experiment (n=3 separate wells per group) but represents multiple observations. **b)** Graph showing oxygen consumption rate (OCR) over time in peritoneal tissue-resident macrophages (Res). Addition of zymosan (50µg/ml) or phorbol-myristate-acetate (PMA, 1µM) to Res results in a burst in OCR unseen in BMDM. Data (n=5 separate ells per group) represent many observations of non-responsiveness in BMDM. Error bars denote mean±SEM.

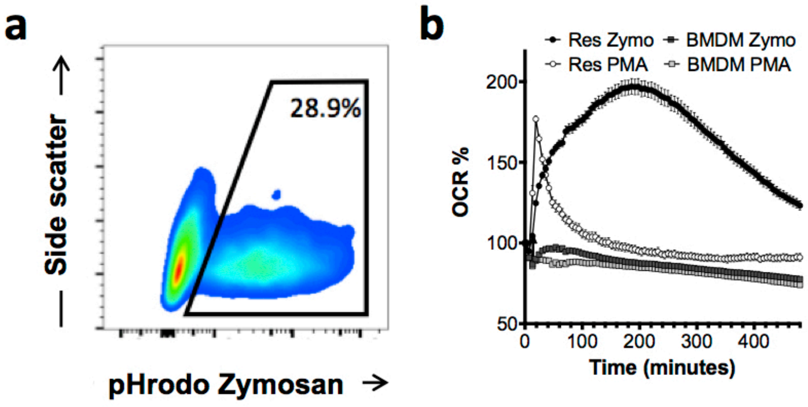

Supplementary Figure 8

Peritoneal tissue-resident macrophages do not make nitric oxide in response to zymosan or *Saccharomyces cerevisiae*

Bar graph showing the log of peritoneal tissue-resident macrophage nitrite production levels 24h after zymosan (50µg/ml) or *Saccharomyces cerevisiae (S.cer.)* (1:2 ratio to macrophages). The LPS on the axis depicts typical production of bone marrow-derived macrophage nitrate 24h after 100ng/ml LPS. Data (n=3-4 per group) are from two experiments and represent at least two independent repeats. Error bars denote mean±SEM.

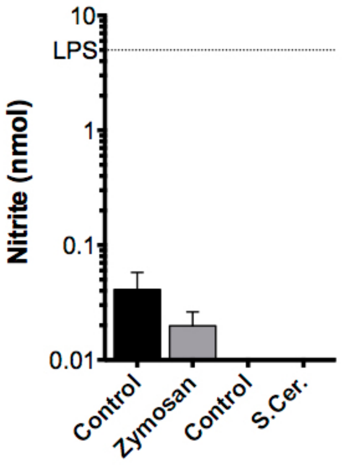

Supplementary Figure 9

Peritoneal tissue-resident macrophages lacking TLR2 localize their mitochondria to the phagolysosome

Graphs showing quantification of the number of Mitotracker positive pixels expressed as a percentage of total (termed mitochondrial localization %) at different radial distances from zymosan particles. Data shown are from zymosan particles ingested in 37 wild-type (WT) and 31 toll-like receptor 2 knockout (TLR2 KO) peritoneal tissue-resident macrophages. Data were from at least 5 separate images and were analysed by two-way ANOVA (Interaction $p<0.0001$, distance $p<0.0001$, genotype $p>0.9999$). Linear regression analysis is additionally depicted on the graphs. Whiskers show 10-90% of the data range.

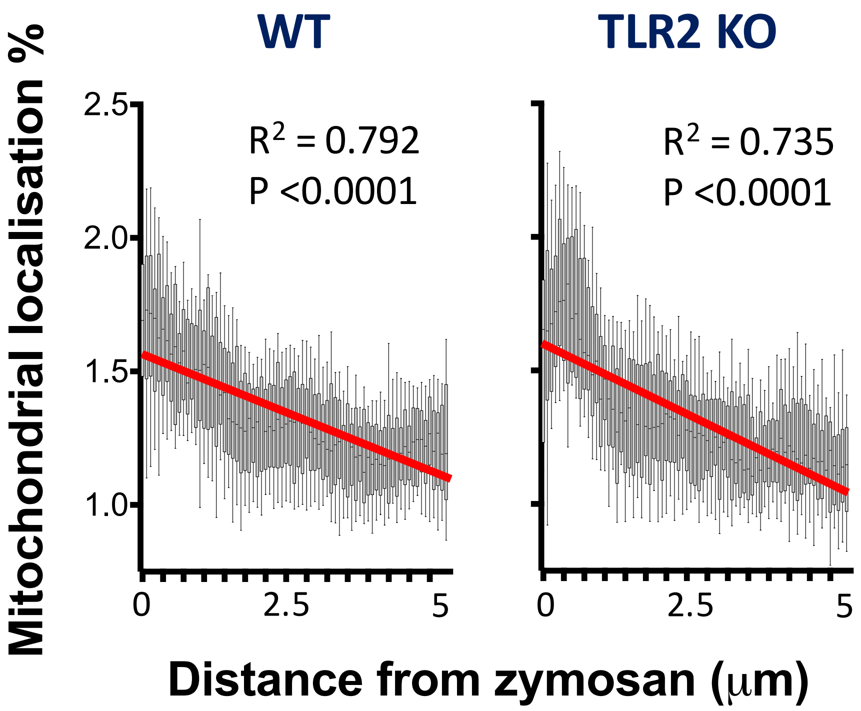

Supplementary Figure 10

Flow cytometry gating strategies

a) Representative flow cytometry density plots showing gating on singlets followed by gating of Ly-6G positive neutrophils in samples after positive selection with Ly-6G magnetic bead labelling (FITC=fluorescein isothiocyanate). **b)** Representative flow cytometry density plots showing gating on singlets followed by gating of F4/80 positive peritoneal resident macrophages in samples after positive selection with F4/80 magnetic bead labelling (APC=allophycocyanin). For a and b, CD11b APC-Cy7 was also used to confirm positive selection. **c)** Representative flow cytometry density plots showing gating on singlets followed by gating of apoptotic (AnnexinV$^+$, 7AAD$^-$) and dead (AnnexinV$^+$, 7AAD$^+$) thymocytes after 3h treatment with dexamethasome (1µM).

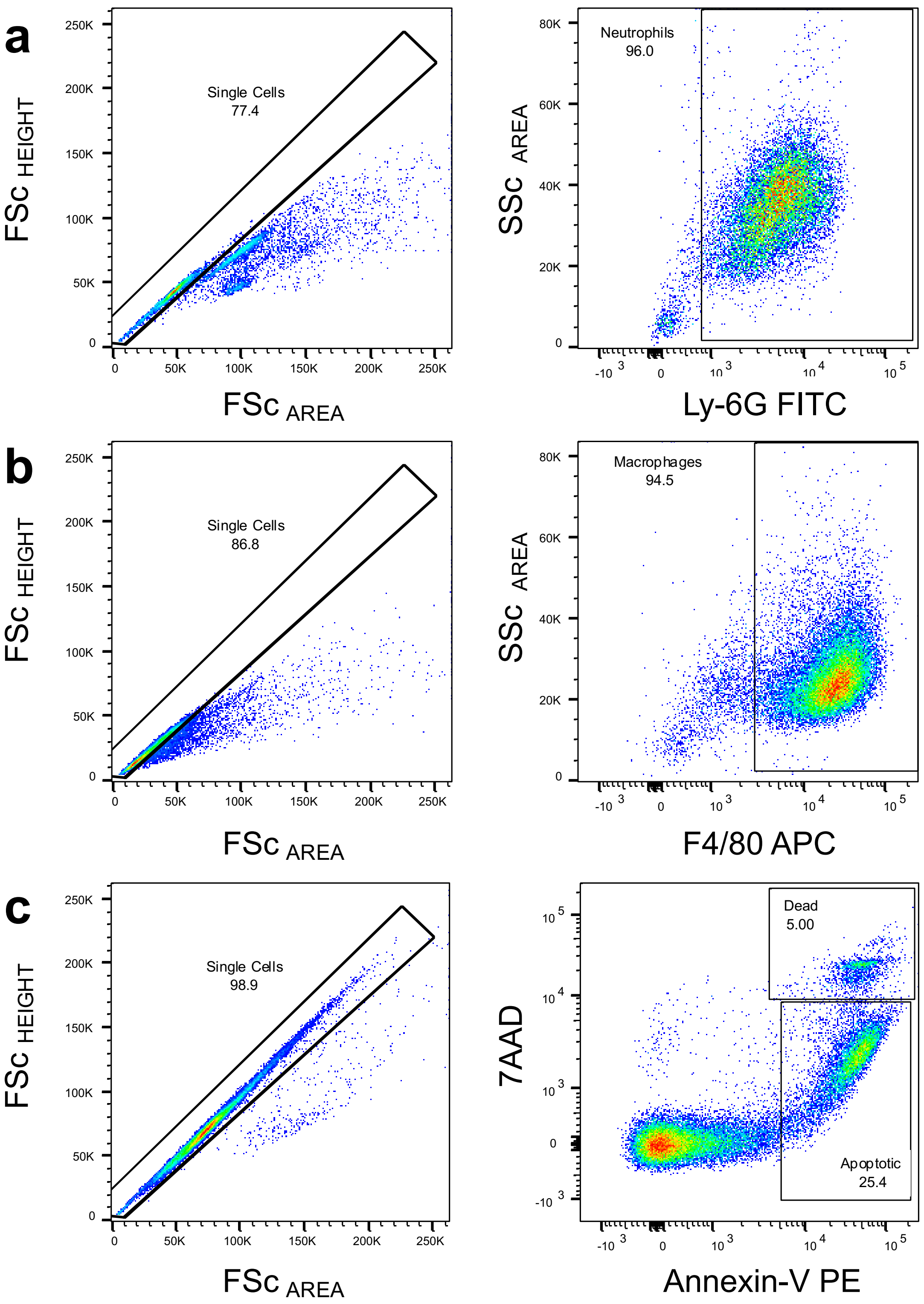
a
Single Cells
77.4
FSc HEIGHT
FSc AREA
Neutrophils
96.0
SSc AREA
Ly-6G FITC
b
Single Cells
86.8
FSc HEIGHT
FSc AREA
Macrophages
94.5
SSc AREA
F4/80 APC
c
Single Cells
98.9
FSc HEIGHT
FSc AREA
Dead
5.00
Apoptotic
25.4
7AAD
Annexin-V PE

Supplementary Figure 11

Trypan blue dilution for evaluation of peritoneal fluid volume

Graph showing a dilution of trypan blue absorbance (590nm) with phosphate buffered saline (mock peritoneal fluid volume). The dotted lines represent actual peritoneal lavages with trypan blue (n=4), which record a peritoneal volume of 98.18±5.69 (mean±standard error of the mean) by trypan dilution. The data represent at least two independent experiments.

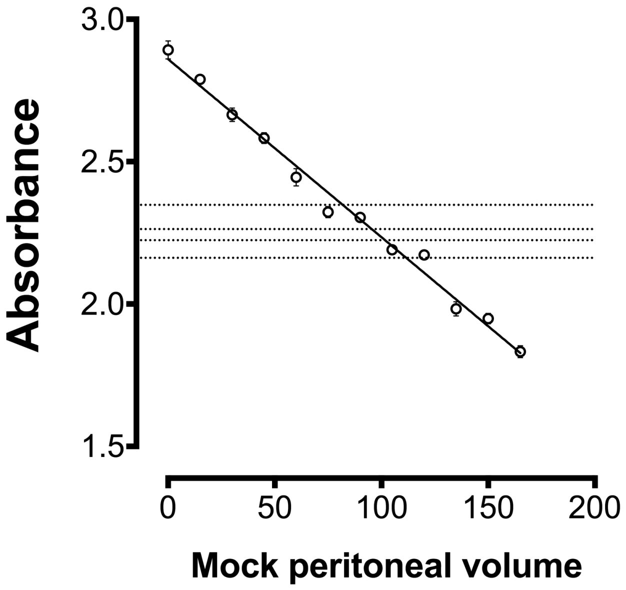

Supplement: Supplementary file 1 — Supplementary Information [file 41467_2017_2092_MOESM1_ESM.pdf]
