# Supplementary material for: Peritoneal tissue-resident macrophages are metabolically poised to engage microbes using tissue-niche fuels
Source: Nat Commun. 2017 Dec 12;8:2074. doi: 10.1038/s41467-017-02092-0 (PMC5727035; doi:10.1038/s41467-017-02092-0)
Supplement: Supplementary file 3 — Description of Additional Supplementary Files [file 41467_2017_2092_MOESM3_ESM.pdf]

Description of Additional Supplementary Files

File Name: Supplementary Movie 1
Description: **Raw bone marrow-derived macrophage mitochondrial volume**
Video showing mitochondria from a single bone marrow-derived macrophage in greys. Mitochondria were labeled with MitotrackerRed CMXRos (25nM). Data represents at least 9 images, pooled from two independent observations (n=30).

File Name: Supplementary Movie 2
Description: **Modelled bone marrow-derived macrophage mitochondrial volume**
Video showing mapped mitochondrial volume from a single bone marrow-derived macrophage (in Supplementary Video 1). Mitochondria were labeled with MitotrackerRed CMXRos (25nM), and raw data were analyzed with the 3D object counter in Fiji. Visually Fig.SV1 matches this model. Data represent at least 9 images, pooled from two independent observations (n=30).

File Name: Supplementary Movie 3
Description: **Raw peritoneal tissue-resident macrophage mitochondrial volume**
Video showing mitochondria from a single peritoneal tissue-resident macrophage in greys. Mitochondria were labeled with MitotrackerRed CMXRos (25nM). Data represent at least 9 images, pooled from two independent observations (n=34).

File Name: Supplementary Movie 4
Description: **Modelled peritoneal tissue-resident macrophage mitochondrial volume**
Video showing mapped mitochondrial volume from a single peritoneal tissue-resident macrophage (in Supplementary Video 3). Mitochondria were labeled with MitotrackerRed CMXRos (25nM), and raw data were analyzed with the 3D object counter in Fiji. Visually Fig.SV1 matches this model. Data represent at least 9 images, pooled from two independent observations (n=34).

File Name: Supplementary Data 1
Description: Peritoneal tissue-resident macrophage vs bone marrow-derived macrophage metabolomics

File Name: Supplementary Data 2
Description: Peritoneal fluid lavage vs serum metabolomics
